# Periodontitis and gingival bleeding associate with intracranial aneurysms and risk of aneurysmal subarachnoid hemorrhage

**DOI:** 10.1007/s10143-019-01097-1

**Published:** 2019-04-10

**Authors:** Joona Hallikainen, Antti Lindgren, Jarno Savolainen, Tuomas Selander, Antti Jula, Matti Närhi, Timo Koivisto, Jari Kellokoski, Pekka Ylöstalo, Anna Liisa Suominen, Juhana Frösen

**Affiliations:** 1 Department of Dentistry, University of Eastern Finland, Kuopio, Finland; 2 Hemorrhagic Brain Pathology Research Group, Kuopio University Hospital, Kuopio, Finland; 3 Department of Neurosurgery, Kuopio University Hospital, Puijonlaaksontie 2, 70210 Kuopio, Finland; 4 Department of Oral and Maxillofacial Diseases, Kuopio University Hospital, Kuopio, Finland; 5 Science Service Center, Kuopio University Hospital, Kuopio, Finland; 6 National Institute for Health and Welfare, Helsinki, Finland; 7 Department of Biomedicine, University of Eastern Finland, Kuopio, Finland; 8 Unit of Oral Health Sciences, University of Oulu, Oulu, Finland; 9 Medical Research Center Oulu, Oulu University Hospital, Oulu, Finland

**Keywords:** Intracranial aneurysm, Subarachnoid hemorrhage, Periodontitis, Gingivitis, Risk of rupture

## Abstract

Oral bacteria DNA has been found in intracranial aneurysms (IA) and a high prevalence of periodontitis was reported in IA patients. We investigated whether periodontitis associates with IA formation and aneurysmal subarachnoid hemorrhage (aSAH). First, we compared in a case-control setting the prevalence of periodontal disease in IA patients (42 unruptured IA, 34 ruptured IA) and in age- and gender-matched controls (*n* = 70) from the same geographical area (Health 2000 Survey, BRIF8901). Next, we investigated whether periodontitis at baseline associated with aSAH in a 13-year follow-up study of 5170 Health 2000 Survey participants. Follow-up data was obtained from national hospital discharge and cause of death registries. Univariate analysis, logistic regression, and Cox-regression were used. Periodontitis (≥ 4mm gingival pocket) and severe periodontitis (≥ 6mm gingival pocket) were found in 92% and 49% of IA patients respectively and associated with IAs (OR 5.3, 95%CI 1.1–25.9, *p* < 0.000 and OR 6.3, 95%CI 1.3–31.4, *p* < 0.001, respectively). Gingival bleeding had an even stronger association, especially if detected in 4–6 teeth sextants (OR 34.4, 95%CI 4.2–281.3). Severe periodontitis in ≥ 3 teeth or gingival bleeding in 4–6 teeth sextants at baseline increased the risk of aSAH during follow-up (HR 22.5, 95%CI 3.6–139.5, *p* = 0.001 and HR 8.3, 95%CI 1.5–46.1, *p* = 0.015, respectively). Association of periodontitis and gingival bleeding with risk of IA development and aSAH was independent of gender, smoking status, hypertension, or alcohol abuse. Periodontitis and gingival bleeding associate with increased risk for IA formation and eventual aSAH. Further epidemiological and mechanistic studies are indicated.

**Electronic supplementary material:**

The online version of this article (10.1007/s10143-019-01097-1) contains supplementary material, which is available to authorized users.

## Introduction

Intracranial aneurysms (IA) are pathological dilatations in cerebral arteries that may rupture causing devastating aneurysmal subarachnoid hemorrhage (aSAH). Although IAs are common with a prevalence of 2–3% [1] in the adult population, aSAH caused by IA rupture is a relatively rare event (incidence approximately 10/100,000/year) [2]. Even in lifelong follow-up, only 29% of untreated aneurysms rupture [3]. Nonetheless, due to the high mortality (40%) and morbidity associated with aSAH, unruptured IAs are frequently treated by endovascular or microsurgical procedures [4]. Both of these current treatment options are, however, associated with risk of serious complications (5–7%), including death [5, 6]. There is therefore a need for reliable strategies to estimate rupture risk and avoid unnecessary treatments.

Since most unruptured IAs are asymptomatic, it is important to understand the pathobiology of IAs in order to target screening studies and interventions to persons at risk. Many IA patients do not have any of the currently recognized risk factors for IA development and many aSAH patients do not have recognized risk factors for IA rupture [7], suggesting important risk factors remain to be identified. In animal models, IA formation is mediated by inflammation of the artery [8, 9] and inflammation also associates with rupture in human IA walls [10]. Oral bacterial DNA has been found in both ruptured (58–69%) and unruptured IA walls (71%) [11, 12], suggesting oral infections may contribute to IA wall inflammation, especially since oral infections have been previously shown to accelerate inflammation in the wall of other types of aneurysms [13, 14]. Furthermore, a recent epidemiological study reported possible association between increased risk of aSAH and the presence of a specific strain of Streptococcus mutans bacteria in the oral cavity [15], suggesting oral infections might indeed predispose IAs to rupture and subsequent aSAH.

Periodontitis, a chronic inflammatory disease of tooth-supporting tissues, associates with the development of abdominal aortic aneurysms (AAA) [13, 14], where it accelerates inflammation [14]. A relatively high incidence of severe periodontitis (as defined by the presence of gingival pockets ≥ 6 mm deep) has recently been reported for a case series of 90 IA patients admitted for treatment [16]. Since prior literature suggests oral infections and especially periodontitis might predispose patients to the formation and progression of IAs, as it does for extracranial aneurysms [13, 14], we investigated whether periodontitis or clinically evident gingival inflammation is an independent risk factor for either IA formation or aSAH.

## Material and methods

This study was performed in three sequential steps: first was a case series study of the prevalence of periodontitis and gingival inflammation in IA patients, second was a case-control comparison of these same IA patients with age- and gender-matched controls from the same geographical area, and third was a prospective follow-up of persons affected by periodontitis or gingival inflammation. The overall aims of these steps were to (1) confirm previously reported high prevalence of periodontitis among IA patients, (2) investigate in a case-control setting whether periodontitis or gingival inflammation associates with IA formation, independent of other known risk factors for IA formation or rupture, and (3) investigate whether periodontitis or gingival inflammation not only associates with IA formation but also with aSAH, the clinical end-point that treatment and clinical management of IA patients are designed to prevent. This study was approved by the ethical review boards of the Healthcare District of Northern Savo (patient recruitment and data collection at Kuopio University Hospital (KUH)) and The Ethical Committee for Research in Epidemiology and Public Health at the Hospital District of Helsinki and Uusimaa in Finland (Health 2000 [17] and 2011 Surveys [22]). Written informed consent was obtained from all participants.

### Study population for the case series of IA patients

**Fig. 1:**
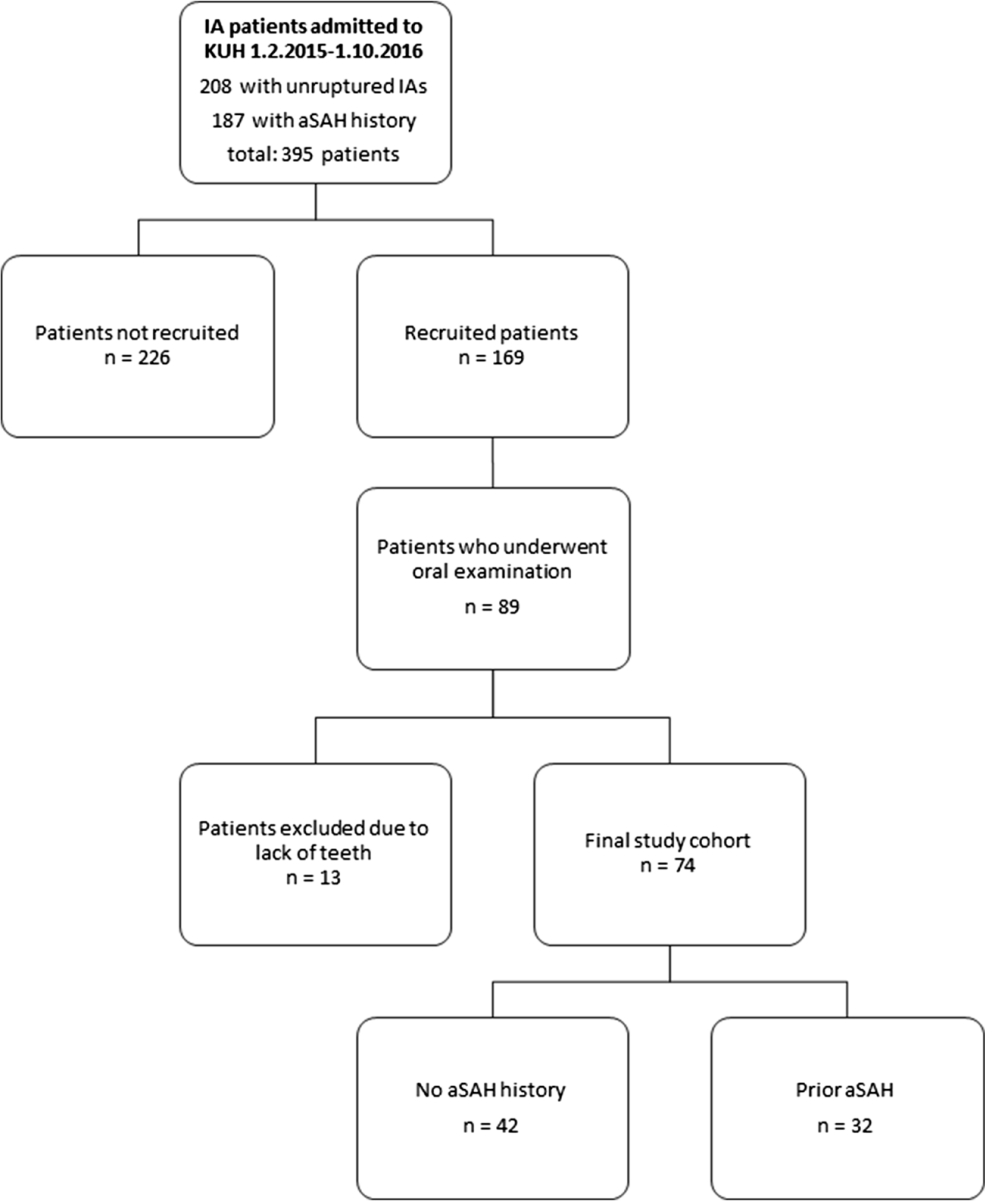
Flow chart demonstrating the recruitment of KUH IA patients

Patients referred to the Department of Neurosurgery of KUH for IA treatment during the period between February 1st 2015 and October 1st 2016 were recruited to this study. Ultimately, a clinical oral examination was performed as described below on 89 IA patients (42 with unruptured IAs and 34 with aSAH) selected according to the availability of an examining dentist. Thirteen patients were excluded due to complete loss of natural teeth (Fig. 1). In addition, two patients were excluded from statistical analysis due to an insufficient oral examination. For the KUH IA patients, data including known risk factors for both aSAH [18, 19] and IA formation (including periodontitis[20]) were collected from the medical reports and a personal interview.

### Case-control comparison of IA patients with matched controls

**Fig. 2:**
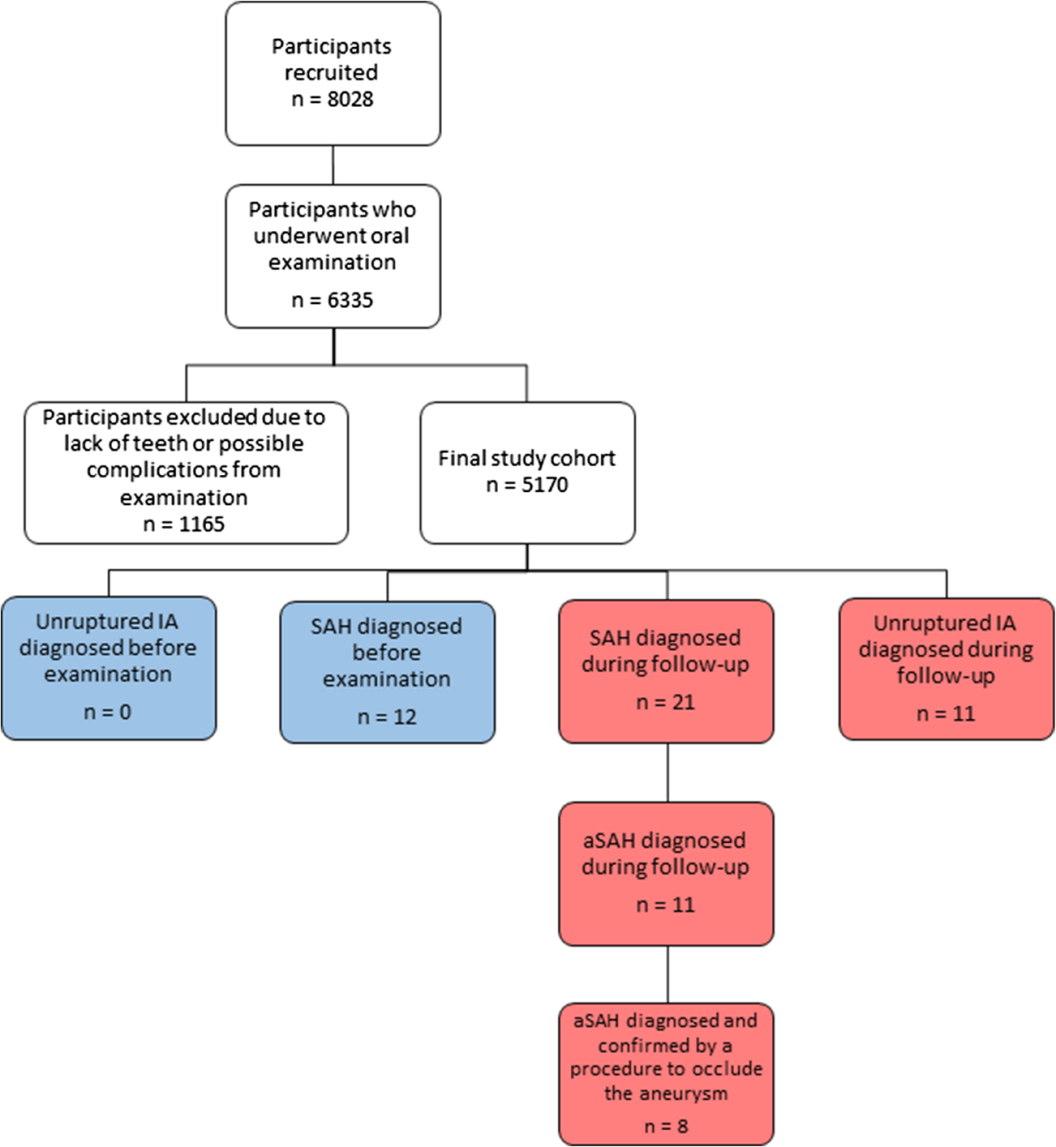
Flow chart demonstrating the selection of the Health 2000 Survey participants included in this study

The prevalence and severity of periodontitis among the KUH IA patients were compared with a previously published prospectively collected epidemiological data from a cohort of 8028 adult Finns, of whom 6771 participated in a comprehensive health survey (Health 2000 Survey) [17]. After exclusion of persons with no teeth or in whom periodontal examination could not be properly performed in all remaining teeth, 5170 patients remained and were included in this study following baseline clinical oral examination (Fig. 2, supplementary data Table S1 and S2). For the Health 2000 Survey participants, data of known risk factors for aSAH or IA development (gender, smoking, hypertension, alcohol abuse) [18, 19], as well as known risk factors for periodontitis (smoking, diabetes, alcohol abuse) [20, 21], were collected using a questionnaire during the initial study enrolment and clinical examination, as described previously [17]. To identify possible prior aSAH or unruptured IA diagnosed before baseline examination that had been unreported by the study participants, the national registry for hospital discharge diagnosis (HILMO or Care Register for Health Care), maintained by the National Institute for Health and Welfare in Finland, was searched for each of the Health 2000 Survey participants for the diagnoses with ICD-10 codes I67.1 or I60.0-I60.9, as well as for surgical or endovascular procedures that are related to treatment of intracranial aneurysms and classified according to the Nordic Classification for Surgical Procedures (supplementary data Table S1).

Age- and gender-matched controls without a history of previously diagnosed aSAH or unruptured IA were identified among the Health 2000 Survey participants from the region of Eastern Finland that corresponds to the KUH catchment area. Logistic regression was used to compare the KUH IA patient series with these age- and gender-matched controls from the same geographical area.

### Prospective follow-up of Health 2000 Survey participants for aSAH

Follow-up data for patients with aneurysmal SAH diagnosed after the baseline Health 2000 Survey, but before December 31st 2013, were collected from the national registry for hospital discharge diagnosis (HILMO or Care Register for Health Care) as described above. The diagnosis suggesting aSAH obtained from the hospital discharge registry was confirmed as true aSAH by cross checking the same registry for performed procedures related to the treatment of aSAH and classified according to the Nordic Classification for Surgical Procedures (supplementary data Table S1). Cox-regression was used to calculate the hazard ratio for suffering aSAH during the 13-year follow-up after the baseline examination. In addition, all participants of the Health 2000 Survey were re-invited to a follow-up health survey in 2011 (Health 2011 Survey) [22], which included a second oral examination for those that were residents of Southern or Northern Finland. Data of the oral examination in the Health 2011 Survey was compared to the status 11 years earlier (Health 2000 Survey) for those participants affected by aSAH during the time interval between the studies, and for whom data from both studies were available.

### Clinical examination for periodontitis

Periodontal status was registered in both KUH IA patients and in Health 2000 Survey participants according to the Health 2000 Survey protocol by measuring the periodontal pockets (periodontal probing depth) in all permanent teeth excluding wisdom teeth from six or four points around each tooth (six in KUH patients, four in Health 2000 Survey).[17]

In KUH IA patients, measurements were done with LM 8-520B perio probe with indication marks at every 2 mm and the pocket depths were rounded to the nearest millimeter. In addition, bleeding on probing (BOP), as a marker of ongoing inflammation in gingival tissue, was registered from four sites per tooth and was classified according to the number of tooth sextants presenting bleeding. Periodontitis was diagnosed if ≥ 4 mm probing depth was found in at least one tooth. Severe periodontitis was diagnosed if at least one tooth site had ≥ 6 mm probing depth. Health 2000 Survey participants and KUH IA patients were similarly classified into three categories according to probing depth: < 4 mm no periodontitis, 4–5 mm periodontitis, and ≥ 6 mm severe periodontitis. Deepest periodontal probing depth in each tooth was taken into account.

### Statistical analyses

Data were presented as means with ranges or frequencies with percentages and 95% confidence intervals. Fisher’s exact test or chi-square tests were used to compare categorical variables and Mann-Whitney *U* for continuous variables, as appropriate. Logistic regression and Cox-regression were used for multivariate analysis as described above. Results from both regression analyses were presented as odds- or hazard ratios with 95% confidence intervals. SPSS 22.0 statistical software (IBM) was used and a *p* value < 0.05 was considered significant.

## Results

### High frequency of periodontitis in a case series of intracranial aneurysm patients

Periodontitis was found in 91.9% (68/74) of the KUH IA patient cohort, among whom severe periodontitis was found in 48.6% (36/74) (Table 1). The prevalence of periodontitis was 1.4 times higher (91.9% vs 67.1%) and the prevalence of severe periodontitis 1.9 times higher among KUH IA patients than among the Health 2000 Survey participants that represent the general adult Finnish population (48.6% vs. 25.7%). No significant difference in the prevalence of periodontitis (90.3% vs 94.0%) or severe periodontitis (48.8% vs 48.5%), as determined by periodontal probing depth, was found between KUH IA patients presenting with unruptured and ruptured IAs.

**Table 1:** Demographics, aSAH risk factors, and periodontitis in the studied intracranial aneurysm patients from Kuopio University Hospital (KUH IA patients). Data is presented as median and range or as proportions with 95%CI

Variable	aSAH (*n* = 33)	No aSAH (*n* = 41)	*p* value	Matched controls (*n* = 70)	*p* value
Age	50.0 (27.0–76.0)	60.0 (22.0–77.0)	0.004	57.0 (31.0–76.0)	NS
Gender (number of females)	23/33 (69.7%, 95%CI 51.1–83.8)	25/41 (61.0%, 95%CI 44.6–75.4)	NS	45/70 (64.3%, 95%CI 51.9–75.1)	NS
IA family history	5/33 (15.2%, 95%CI 5.7–32.7)	6/41 (14.6%, 95%CI 6.1–29.9)	NS		
Multiple IAs	17/33 (51.5%, 95%CI 33.9–68.8)	17/41 (41.5%, 95%CI 26.7–57.8)	NS		
Hypertension (treated with medication)	16/33 (48.5%, 95%CI 31.2–66.1)	23/41 (56.1%, 95%CI 39.9–71.2)	NS	40/70 (57.1%, 95%CI 44.8–68.7)	NS
Untreated or inadequately treated hypertension	3/33 (9.1%, 95%CI 2.4–25.5)	0/41	NS		
Diabetes (type I or II)	0/33	3/41 (7.3%, 95%CI 1.9–21.0)	NS		
Heavy alcohol consumption (defined by treating physician)	1/33 (3.0%, 95%CI 0.2–17.5)	3/41 (7.3%, 95%CI 1.9–21.0)	NS		
Current smoking	16/33 (48.5%, 95%CI 31.2–66.1)	10/41 (24.4%, 95%CI 12.9–40.6)	0.047	26/70 (37.1%, 95%CI 26.1–49.6)	NS
No smoking history	16/33 (48.5%, 95%CI 31.2–66.1)	23/41 (56.1%, 95%CI 39.9–71.2)	0.047		
Periodontitis (≥ 4 mm periodontal pocket)	31/33 (93.9%, 95%CI 78.4–98.9)	37/41 (90.2%, 95%CI 75.9–96.8)	NS	47/70 (67.1%, 95%CI)	< 0.001
Periodontitis (periodontal pocket 4–5 mm)	15/33 (45.5%, 95%CI 28.5–63.4)	17/41 (41.5%, 95%CI 26.7–57.8)	NS NS	29/70 (41.4%, 95%CI 30.0–53.8)	< 0.001
Severe periodontitis (≥ 6 mm periodontal pocket)	16/33 (48.5%, 95%CI 31.2–66.1)	20/41 (48.8%, 95%CI 33.2–64.6)		18/70 (25.7%, 95%CI 16.3–37.8)	< 0.001
Mean BOP %	40.1%	37.9%	NS		
Number of bleeding sextants					
0–1	1/33 (3.0%, 95%CI 0.2–17.5)	2/41 (4.9%, 95%CI 0.9–17.8)	NS	39/70 (55.7%, 95%CI 43.4–67.4)	< 0.001
2–3	6/33 (18.2%, 95%CI 7.6–36.1)	11/41 (26.8%, 95%CI 14.8–43.2)	NS	18/70 (25.7%, 95%CI 16.3–37.8)	< 0.001
4–6	26/33 (78.8%, 95%CI 60.6–90.4)	28/41 (68.3%, 95%CI 51.8–81.4)	NS	13/70 (18.6%, 95%CI 10.6–30.0)	< 0.001
Gingival bleeding from teeth sextants (mean sextants affected)	4.8	4.3	NS	1.0	< 0.001

### Periodontitis and gingival inflammation associate with intracranial aneurysms in case-controlled multivariate comparison

Next, we compared KUH IA patients with age- and gender-matched controls from the same geographical area. These controls were Health 2000 Survey participants from KUH catchment area without prior SAH or IA diagnosis. In a logistic regression model that included known risk factors of IA development and aSAH (gender, smoking, hypertension, alcohol abuse), it was found that periodontitis, as determined by periodontal probing depth, was associated with IA development (patients with unruptured or ruptured IAs) (Table 2). Gingival bleeding on probing, a sign of active gingival inflammation that can predispose to periodontitis, was also significantly associated with IA development (Table 2).

**Table 2:** Multivariate analysis of periodontitis and risk factors for IA formation among the studied intracranial aneurysm patients from Kuopio University Hospital (KUH IA patients) and matched controls

Variable	Cases	Odds ratio	95%CI	*p* value
Model 1				
Age	144	0.950	0.912–0.989	0.013*
Gender				
Male	51/144	1		
Female	93/144	1.869	0.690–5.063	0.219
Hypertension				
No confirmed hypertension	62/144	1		
Confirmed hypertension	82/144	1.172	0.469–2.925	0.734
Smoking status				
Non-smoking	74/144	1		
Intermittent smoking	18/144	0.180	0.020–1.621	0.126
Daily smoking	52/144	1.717	0.654–4.504	0.272
Periodontitis*				
No periodontitis	29/144	1		
Periodontitis	61/144	5.315	1.089–25.935	0.039*
Severe periodontitis	54/144	6.312	1.270–31.372	0.024*
Model 2				
Age	144	0.959	0.920–1.000	0.048*
Gender				
Male	51/144	1		
Female	93/144	2.377	0.777–7.268	0.129
Hypertension				
No confirmed hypertension	62/144	1		
Confirmed hypertension	82/144	1.088	0.434–2.728	0.858
Smoking status				
Non-smoking	74/144	1		
Intermittent smoking	18/144	0.228	0.024–2.138	0.195
Daily smoking	52/144	3.045	0.995–9.318	0.051
Gingival bleeding**				
0–1 sextants	42/144	1		
2–3 sextants	35/144	12.475	1.331–116.905	0.027*
4–6 sextants	67/144	34.356	4.196–281.305	< 0.001*

In logistic regression analysis, age, gender, periodontitis determined by depth of periodontal pockets, and gingival bleeding determined by bleeding on probing (BOP) were associated with the presence of unruptured or ruptured IAs (models 1 and 2). Comparisons were made between the KUH IA patient cohort including patients with unruptured (*n* = 41) and ruptured IAs (*n* = 33), and an age- and gender-matched subgroup of Health 2000 Survey participants (*n* = 70) from the same geographical region as the patient referral catchment area of KUH Neurosurgery

*Periodontal probing depth was categorized according to the presence of at least one tooth with ≥ 6 mm probing depth (severe periodontitis), 4–5 mm probing depth (periodontitis), or with no teeth having ≥ 4 mm probing depth (no periodontitis)

**Gingival bleeding was defined as a number of tooth sextants in which bleeding occurred from the gingival margin on probing. Periodontal probing depth and gingival bleeding on probing were not included in the models simultaneously due to high intervariable correlation

### Periodontitis and gingival inflammation at baseline associate with risk of aSAH during follow-up

Overall, prior non-traumatic SAH (ICD-10 codes I60.0-I60.9) had been diagnosed in 12/5170 of the Health 2000 Survey participants at baseline (supplementary data Table S2). Periodontitis was significantly more common among these 12 survey participants than among the rest of the participants (83.3% or 10/12 vs 63.4% or 3269/5158, *p* = 0.017, Fisher), corresponding to our findings with KUH IA patients.

During the 13-year follow-up period from the original survey to December 31st 2013, an additional 21 of the Health 2000 Survey participants received a new diagnosis for non-traumatic SAH (ICD-10 codes I60.0-I60.9, supplementary data Table S3). Of these 21 participants, 11 received a diagnosis code indicating aneurysmal SAH (I60.0-I60.6) and 8 of them also underwent a surgical procedure for the occlusion of an intracranial aneurysm, confirming the aSAH diagnosis (supplementary data, Table S4).

**Fig. 3:**
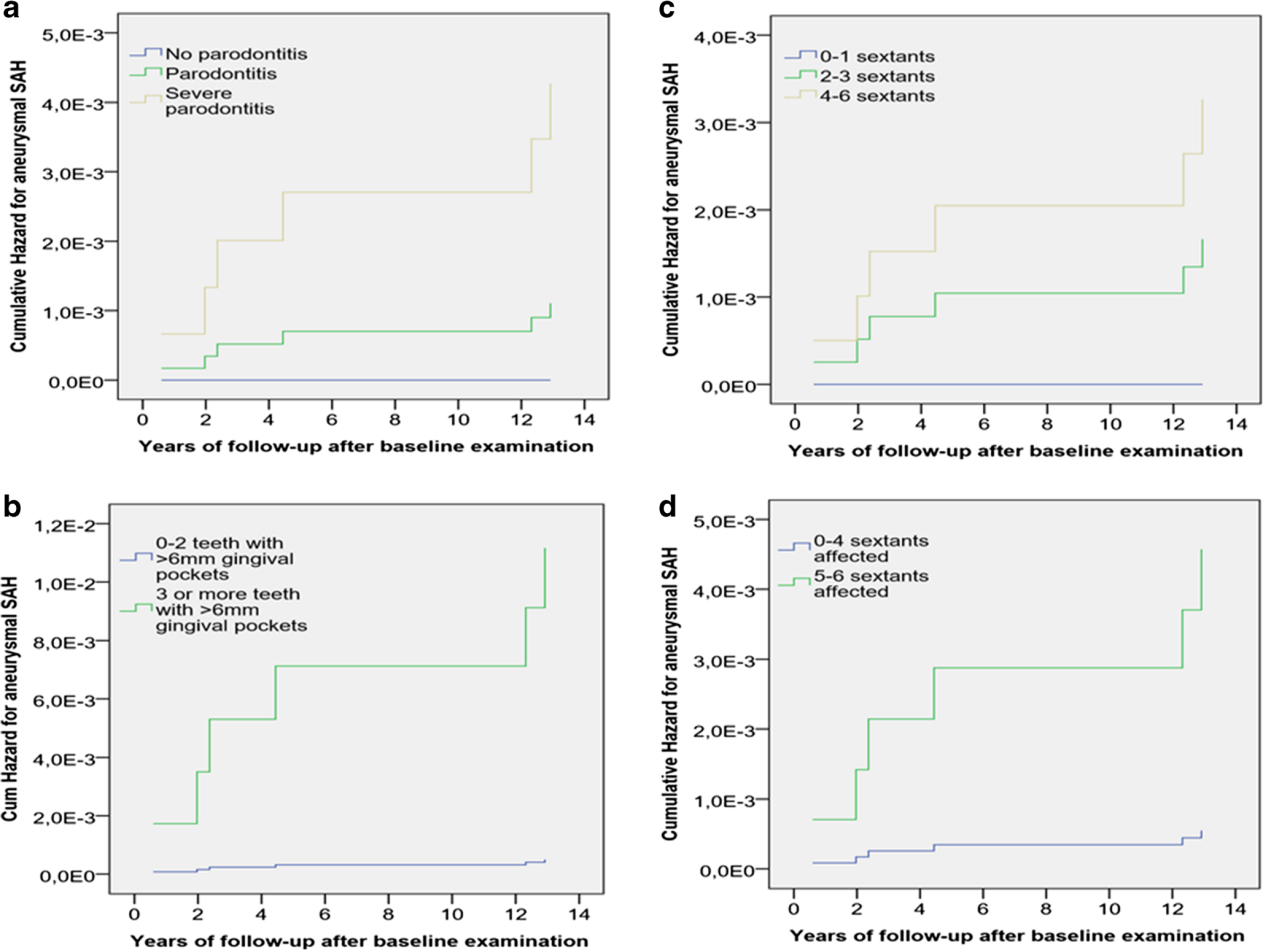
Cumulative hazard for aneurysmal SAH during 13-year follow-up of the Health 2000 Survey participants. Separate lines were plotted for participants with severe periodontitis, periodontitis, and no periodontitis at baseline in **a**, and for participants with 0–2 teeth or ≥ 3 teeth affected by severe periodontitis (≥ 6 mm deep periodontal pocket) in **b**. Similarly, separate lines were plotted in **c** and **d** according to the extent of gingival inflammation, categorized according to the number of teeth sextants affected by gingival infection. The variables included in the Cox-regression are given in Table 3, as well as the corresponding hazard ratios and *p* values

Periodontitis at baseline, as well as gingival inflammation at baseline, was associated with increased risk for diagnosis of aneurysmal SAH during the follow-up (Fig. 3, Table 3, and supplemental data Table S5). When only confirmed aneurysmal SAH diagnosis (supplementary data Table S4) was considered as an end-event, both the extent of severe periodontitis as measured by the number of teeth with ≥ 6 mm periodontal pockets, as well as the extent of gingival inflammation, as measured by the number of sextants with bleeding on probing at baseline, associated with increased risk for aSAH in Cox-regression model including age, gender, hypertension, and smoking as co-variates (Table 3). Having severe periodontitis in three or more teeth increased the risk of aSAH by 22.5 times and having gingival inflammation in more than half of the teeth sextants increased the risk of aSAH by 8.3 times (Table 3). Oral examination was repeated 11 years after the baseline examination in 6/11 of the Health 2000 Survey participants who received an aSAH diagnosis during follow-up, and in 4/6 of them gingival inflammation or periodontitis had progressed (supplemental data Fig. S2).

**Table 3:** Cox-regression analysis of the association of periodontitis and gingival bleeding at baseline with risk of later aneurysmal subarachnoid hemorrhage during follow-up

Variable	Cases in each group/number of followed subjects in the model	Hazard ratio	95%CI	*p* value
Model 1				
Age	2792	1.0	0.9–1.1	0.600
Gender				
Male	1602/2792	1		
Female	1194/2792	4.5	0.8–25.5	0.090
Hypertension				
No confirmed hypertension	1673/2792	1		
Confirmed hypertension	1123/2792	0.6	0.1–3.7	0.575
Smoking status				
Non-smoking	1345/2792	1		
Daily or intermittent smoking	1451/2792	3.6	0.4–32.1	0.256
Severe periodontitis^#^ (≥ 6 mm periodontal pocket)				
0–2 teeth affected	2522/2792	1		
≥ 3 teeth affected	274/2792	22.5	3.6–139.5	0.001*
Model 2				
Age	2786	1.1	1.0–1.1	0.172
Gender				
Male	1598/2786	1		
Female	1189/2786	3.5	0.6–19.6	0.157
Hypertension				
No confirmed hypertension	1668/2786	1		
Confirmed hypertension	1119/2786	0.8	0.1–4.7	0.786
Smoking status				
Non-smoking	1340/2786	1		
Daily or intermittent smoking	1447/2786	5.4	0.6–48.7	0.130
Gingival bleeding^¤^				
0–4 sextants affected	2170/2786	1		
5–6 sextants affected	617/2786	8.3	1.5–46.1	0.015*

In a multivariate analysis that included periodontal probing depth and gingival bleeding as markers of past or ongoing periodontitis and gingival infection, active smoking at the beginning of the follow-up and gingival bleeding on probing were the only significant predictors of aneurysmal SAH during follow-up. The number of followed study participants (cases) with data available on all the included variables were 2792 (model 1) and 2783 (model 2), with 6 of these patients being diagnosed with aSAH during the 13-year follow-up and undergoing a surgical or endovascular procedure for the ruptured IA, thus confirming the diagnosis

Smoking status was categorized as daily or intermittent smoking, or non-smoking. Periodontitis was defined as presence of at least one tooth with ≥ 4 mm probing depth, and severe periodontitis^#^ as the presence of a periodontal pocket ≥ 6 mm deep. Survey participants were categorized according to the number of affected teeth

Gingival bleeding^¤^ was defined as a number of tooth sextants in which bleeding occurred from the gingival margin on probing. Survey participants were categorized according to the number of affected sextants

Periodontal probing depth and gingival bleeding on probing were not included in the models simultaneously due to high intervariable correlation

*Asterisks* are statistically significant *p* values

^#^Also the extent of severe periodontitis as measured by the number of teeth with ≥ 6 mm periodontal pockets at baseline (continuous variable) associated with risk of aSAH (HR 1.2, 95%CI 1.1–1.4, *p* = 0.002)

^¤^Also the extent of gingival inflammation as measured by the number of sextants with bleeding on probing at baseline (continuous variable) associated with increased risk for aSAH (HR 1.7, 95%CI 1.1–2.5, *p* = 0.021)

## Discussion

In addition to prior studies finding DNA of oral bacteria in unruptured and ruptured IA walls [11, 12], a recent study showed an association between being a carrier of a dental cariogenic pathogen Streptococcus mutans with aSAH [15] and another recent case series reported high prevalence of periodontitis in IA patients [16]. The present study is, however, the first to investigate the association of actual clinical periodontitis with the development and rupture of IA in humans with a control study setting. In addition to a case series of IA patients, we performed a case-control multivariate analysis with IA patients and age- and gender-matched controls, as well as a follow-up study of 5170 health survey participants. In this study, the prevalence of periodontitis was significantly higher in IA patients than in the general adult population (both in KUH IA patient series as well as at the baseline examination of the Health 2000 Survey) but did not differ among patients with ruptured or still unruptured aneurysms. This suggests that periodontitis is a risk factor for IA development, and as such a risk factor for aSAH. Our follow-up study of Health 2000 Survey participants confirmed that indeed active gingival inflammation, a precursor of periodontitis, associates with increased risk of later IA rupture and subsequent aSAH.

Together our results imply that infection and inflammation of the gingival and periodontal tissues are a novel risk factor for IA development, progression, and subsequent aSAH. Though this gingival inflammation and periodontitis associated increase in risk of aSAH seems to be mediated by predisposition to develop IAs, we cannot exclude that progression of the periodontitis and gingival inflammation could also promote the inflammatory reaction observed in the walls of unruptured IAs and thus also predispose to progression of unruptured IAs towards rupture.

Gingivitis and periodontitis are bacteria-driven chronic inflammatory diseases that can be separated by localization. Gingivitis is inflammation on gingival tissue, whereas periodontitis affects also the tooth-supporting tissues. Without adequate treatment gingivitis, in many cases, progresses to periodontitis causing destruction of the tooth-supporting ligaments and alveolar bone of the jaws. Periodontal pathogens can disseminate to extra-oral sites via circulation when transient bacteremia is induced by dental procedures, or even by tooth brushing or chewing in cases of severe gingival or periodontal infection [23, 24]. Through this transient bacteremia, gingivitis and periodontitis can also affect the systemic inflammatory response. Previously periodontitis has been associated with cardiovascular diseases such as stroke [25, 26]. Moreover, periodontitis has been shown to associate with remodelling of the aneurysm wall in AAA. Additionally, in experimental models, periodontal bacteria promote AAA wall degeneration by increasing the recruitment of neutrophils to the intraluminal thrombus covering the inner part of AAA [14]. IA and AAA pathology share many features, for example, for both neutrophils are attracted by the intraluminal thrombus [27, 28]. Pyysalo M et al. first demonstrated the presence of oral bacteria DNA in the walls of both ruptured and unruptured IA walls [11, 12], which raises the intriguing question whether oral bacteria can cause or modulate the inflammation observed in an intracranial aneurysm wall [10, 29, 30]. Inflammation of the cerebral artery wall plays a crucial role in the development of intracranial aneurysms [31–33], and it was recently found that modulation of macrophage activity and systemic inflammatory response [9, 32, 33] reduce IA development and progression. Since periodontitis can accelerate activation and recruitment of circulating neutrophils or monocytes and lead to an overall activation of the inflammatory system, it might change the course of cerebral artery remodelling and aneurysm pathology in a way that predisposes the artery towards aneurysm development and rupture.

### Possible biological interaction between smoking and periodontitis as risk factors for IA development and aneurysmal SAH

Smoking is an established risk factor for aSAH [18, 19], and therefore not surprisingly a significant risk factor for aSAH in this study. In spite of smoking also being a risk factor for periodontitis [20], it is important to note that periodontitis and gingival inflammation were independently associated with IA development and rupture (Tables 1, 2, and 3), especially in the KUH IA patients (Tables 1 and 2). This demonstrates that gingival infection and periodontitis are not just co-variates associated with smoking, but indeed novel risk factors for IA development and rupture. Heavy alcohol consumption is also a common risk factor for both periodontitis [21] and aSAH [19], although, as for smoking, it cannot explain the association of periodontitis and gingival inflammation with aSAH and unruptured IAs found in our study, since very few (only one aSAH and four unruptured IA patients, Table 1) of our KUH IA patients were heavy drinkers despite > 90% of them having at least mild periodontitis (Table 1). Although the definition we used for heavy drinking, that is a clinical diagnosis for alcohol abuse by the treating physician, might have led to an underestimation of the true frequency of alcohol consumption among our study participants, it is important to note that the degree of alcohol consumption that is clearly a risk factor for aSAH requires substantial drinking (> 150 g/week, or > 12 drinks/week [19]), which is unlikely to remain unnoticed by the treating physician.

The biological mechanisms through which smoking increases the risk of aSAH are unknown. Active smoking and gingival bleeding at baseline were both risk factors for aSAH during the 13 years of follow-up. When included in the same multivariate model, non-smoking reduced the risk for aSAH (Supplementary data Table S3). Thus, it seems possible that at least some of the biological effects of smoking as an aSAH risk factor are related to progression of periodontal disease. This hypothesis is supported by our observation that periodontitis had progressed during the 11-year interval among four of those six participants who received a diagnosis of aSAH during follow-up and underwent a second oral examination, and that five of these participants smoked (Fig. S1 and S2). Together with smoking, alcohol consumption associated with increased risk for periodontitis [34], and thus, it seems possible that at least some of the biological effects of heavy drinking as a risk factor for aSAH might be related to progression of periodontal diseases, similarly to smoking.

### Strengths and limitations

By combining data from our KUH IA patient cohort and a larger population-based Health 2000 survey, our study identified an association between periodontal diseases, IA formation, and eventual aneurysmal SAH in two separate independent study groups. Furthermore, through a data search from national healthcare registries, we were able to generate follow-up data after the initial baseline survey for study participants with prospectively studied oral status. Through this study setting, we were able to reach sufficient statistical power to demonstrate that periodontitis and gingival inflammation detected in a robust clinical examination are indeed associated with increased risk for a relatively rare disease event such as aneurysmal SAH.

This study also has some limitations due to its design. Because the participation of a KUH IA patient to this study required the concomitant presence of the patient, a trained person recruiting the patient to the study (after informed consent), and an examining dentist, in addition to the patient’s willingness to undergo an oral examination, only 1/5 of the IA patients treated at our institution during the study period could be included in the final study cohort (Fig. 1). Although one could speculate that patients with dental infections would have been more eager to participate, we noticed that it was in fact the other way around with those IA patients that had severe dental problems being more likely to decline participation. Thus, the possible bias would in fact rather lead to an underestimation of the prevalence of gingival inflammation and periodontitis in our results. A second possible source of bias is the comparison of KUH IA patients with participants of Health 2000 survey, performed 15–16 years prior. It should be noted, however, that given the mean age of the participants of the survey and the mean age of the KUH IA patients (Table 1), many of them lived at the same time in the same environment, and thus can be regarded as environment and exposure-matched controls rather than as historical controls.

A third possible source of error is that the national registries for hospital discharge diagnosis and for causes of death that were used to generate the follow-up data, are administrative databases, and the data collected originates from multiple sources with potentially erroneous diagnostic coding. To control this source of error, we also collected data for surgical and endovascular procedures performed for IAs, and used that data to confirm the diagnosis of aSAH in 8 of those 11 patients who had a diagnosis of aSAH, thus reducing the risk of false positives and subsequent type I error. It is very unlikely that a person would have been miscoded both for the diagnosis of aSAH and for a surgical or endovascular procedure to treat an intracranial aneurysm. Moreover, our data from the KUH IA patient cohort demonstrates a clear association of periodontal and gingival inflammation with IAs that were confirmed with angiography.

Another source of error in the diagnostic coding for aSAH arises from the fact that the SAH is commonly diagnosed before the ruptured IA is identified, and therefore, the first diagnosis entered to and remaining in the registry might be that of SAH without identified IA (I60.9) despite the aneurysm having been diagnosed later. To reduce the risk of false negatives and subsequent type II error, we also calculated the statistics using all I60 diagnosis codes (including I60.9) as end events (supplemental data) and observed similar results as with the confirmed aSAH diagnosis.

Finally, a single person performed the oral examination in our KUH IA patient cohort (J.H.) which could have led to over- or underestimation of the extent and severity of periodontitis. The prevalence of periodontitis in our IA patient cohort matches, however, the prevalence of periodontitis in diagnosed aSAH patients in the cross-sectional Health 2000 Survey performed by several examiners, suggesting correct diagnostics of periodontitis. Intra- and interexaminer agreement in Health 2000 Survey varied between 77 and 83% [17].

## Summary

We report that periodontitis and gingival bleeding associate with increased risk for IA formation and for subsequent aneurysmal SAH, which is a significant cause of mortality, disability, and loss of productive life years. This finding may explain, at least in part, the previously established role of smoking as a risk factor for aneurysmal SAH. Our findings strongly encourage further epidemiological and mechanistic studies on the topic, also in other populations.

## Electronic supplementary material

ESM 1 (DOCX 1820 kb)
