# Supplementary material for: Periodontitis and gingival bleeding associate with intracranial aneurysms and risk of aneurysmal subarachnoid hemorrhage
Source: Neurosurg Rev. 2019 Apr 10;43(2):669–79. doi: 10.1007/s10143-019-01097-1 (PMC7186244; doi:10.1007/s10143-019-01097-1)
Supplement: Supplementary file 1 — (DOCX 1820 kb) [file 10143_2019_1097_MOESM1_ESM.docx]

Supplementary data

Hallikainen J et al. **“****Periodontitis and gingival bleeding associate with intracranial aneurysms and risk of aneurysmal subarachnoid hemorrhage”**

**Table S1. International Classification for Diseases (ICD)-10 codes related to intracranial aneurysms and codes used for surgical or endovascular treatment of intracranial aneurysms according to the Nordic Classification for Surgical Procedures (NCSP). For the Health 2000 Survey participants, these codes were search in the national registries** for hospital discharge diagnosis (HILMO or Care Register for Health Care) and for causes of death.

| **Classification** | **Code** | **Significance** |
| --- | --- | --- |
| **ICD-10** | **I67.1** | **Unruptured intracranial aneurysm** |
|  | **I60.0** | **Subarachnoid haemorrhage from an aneurysm of the internal carotid artery** |
|  | **I60.1** | **Subarachnoid haemorrhage from an aneurysm of**  **the middle cerebral artery** |
|  | **I60.2** | **Subarachnoid haemorrhage from an aneurysm of**  **the anterior communicating artery** |
|  | **I60.3** | **Subarachnoid haemorrhage from an aneurysm of**  **the posterior communicating artery** |
|  | **I60.4** | **Subarachnoid haemorrhage from an aneurysm of**  **the basilar artery** |
|  | **I60.5** | **Subarachnoid haemorrhage from an aneurysm of**  **the vertebral artery** |
|  | **I60.6** | **Subarachnoid haemorrhage from an aneurysm of**  **another intracranial artery** |
|  | **I60.7** | **Subarachnoid haemorrhage from an aneurysm of**  **an unspecified intracranial artery** |
|  | **I60.8** | **Other non traumatic subarachnoid haemorrhage** |
|  | **I60.9** | **Non traumatic subarachnoid haemorrhage, unspecified** |
| **NCSP** |  |  |
|  | **AAC00** | **Microsurgical ligation of an intracranial aneurysm** |
|  | **AAC05** | **Microsurgical ligation of the feeding artery of an intracranial aneurysm** |
|  | **AAC10** | **Wrapping of the intracranial aneurysm** |
|  | **AAC15** | **Trapping of the intracranial aneurysm** |
|  | **AAC20** | **Intracranial bypass (used concomitantly with aneurysm trapping)** |
|  | **AAL00** | **Endovascular embolization of an intracranial aneurysm** |
|  | **PA2LT** | **Endovascular embolization of an intracranial aneurysm** |
|  | **PA2KT** | **Endovascular embolization of an intracranial aneurysm** |
|  | **PA2MT** | **Endovascular embolization of an intracranial aneurysm** |
|  | **AAF00** | **Ventriculostomy (external ventricular drain)** |

**Table S2. Demographics of Health 2000 participants with non-traumatic SAH (I60.0-I60.9) at baseline.**

| **Variable** | **aSAH (n=12)** | **No aSAH (n=5158)** | **P-value** |
| --- | --- | --- | --- |
| **Age** | **58.00(32.0-74)** | **48.00(30.0-94.0)** | **-** |
| **Gender (number of females)** | **7/12**  **(64.5% 95%CI 52.6-74.9)** | **2736/5158**  **(53.0% 95%CI 51.7-54.4)** | **NS** |
| **Diagnosed Hypertension** | **4/12**  **(33.3% 95%CI 11.3-64.6)** | **2218/5140**  **(43.2% 95%CI 41.8-44.5)** | **NS** |
| **Elevated blood pressure** | **2/12**  **(16.7% 95%CI 2.9-49.1)** | **858/5145**  **(16.7% 95%CI 15.7-17.7)** | **NS** |
| **Diabetes (type I or type II)** | **0/12 (0%)** | **16/5068**  **(0.3% 95%CI 0.2-0.5)** | **NS** |
| **Current smoking**  **Daily**  **Irregular**  **Not smoking** | **10/12**  **(83.3% 95%CI 50.9-97.1)**  **5/10**  **(50,0% 95%CI 20.1-79.9)**  **0/12 (0%)**  **5/10**  **(50,0% 95%CI 20.1-79.9)** | **2811/5158**  **(54.5% 95%CI 53.1-55.9)**  **1147/2811**  **(40.8% 95%CI 39.0-42.7)**  **315/2811**  **(11.2% 95%CI 10.1-12.5)**  **1349/2811**  **(48.0% 95%CI 46.1-49.9)** | **NS**  **NS**  **NS**  **NS** |
| **No prior smoking history** | **1/12**  **(8.3% 95%CI 0.4-40.2)** | **1583/5158**  **(30.7% 95%CI 29.4-32.0)** | **NS** |
| **Periodontitis** | **10/12**  **(83.3% 95%CI 50.9-97.1)** | **3269/5158**  **(63.4% 95%CI 62.1-64.7)** | **0.017** |
| **Severe periodontitis** | **0/12 (0%)** | **1041/5158**  **(20.2% 95%CI 19.1-21.3)** | **-** |
| **Gingival bleeding from teeth sextants (mean for affected sextants)** | **2.3** | **2.4** | **NS** |

| **Number of bleeding sextants:** |  |  |  |
| --- | --- | --- | --- |
| **0** | **3/12**  **(25% 95%CI 6.7-57.2)** | **1386/5158**  **(26.9% 95%CI 25.7-28.1)** | **NS** |
| **1-2** | **5/12**  **(41.7% 95%CI 16.5-71.4)** | **1549/5158**  **(30.0% 95%CI 28.8-31.3)** | **NS** |
| **3-6** | **4/12**  **(33.3% 95%CI 11.3-64.6)** | **2223/5158**  **(43.1% 95%CI 41.7-44.5)** | **NS** |

|  |  |  |  |
| --- | --- | --- | --- |

**Table S3. Demographics of Health 2000 participants who developed non-traumatic SAH (I60.0-I60.9) during follow-up.**

| **Variable** | **aSAH (n=21)** | **No aSAH (n=5149)** | **P-value** |
| --- | --- | --- | --- |
| **Age** | **49.00(31.0-74.0)** | **48.00(30.0-94.0)** | **-** |
| **Gender (females)** | **9/21**  **(42.9% 95%CI 22.6-65.6)** | **2734/5149**  **(53.1% 95%CI 51.7-54.5)** | **NS** |
| **Diagnosed Hypertension** | **10/21**  **(47.6% 95%CI 26.4-69.7)** | **2212/5131**  **(43.1% 95%CI 41.8-44.5)** | **NS** |
| **Elevated blood pressure** | **5/21**  **(23.8% 95%CI 9.1-47.6)** | **855/5136**  **(16.6% 95%CI 15.7-17.7)** | **NS** |
| **Diabetes (type I or type II)** | **0/21 (0%)** | **16/5059**  **(0.3% 95%CI 0.2-0.5)** | **NS** |
| **Known current smoking status**  **Daily**  **Irregular**  **Not smoking** | **15/21**  **(71.4% 95%CI 47.7-87.8)**  **12/15**  **(80.0% 95%CI 51.4-94.7)**  **1/15**  **(6.7% 95%CI 0.4-34.0)**  **2/15**  **(13.3% 95%CI 2.3-41.6)** | **2806/5149**  **(54.5% 95%CI 53.1-55.9)**  **1140/2806**  **(40.6% 95%CI 38.8-42.5)**  **314/2806**  **(11.2% 95%CI 10.1-12.4)**  **1352/2806**  **(48.2% 95%CI 46.3-50.1)** | **0.007**  **0.007**  **0.007**  **0.007** |
| **No prior smoking history** | **3/21**  **(14.3% 95%CI 3.8-37.4)** | **1583/5130**  **(30.9% 95%CI 29.6-32.2)** | **NS** |
| **Periodontitis** | **16/21**  **(76.2% 95%CI 52.5-90.9)** | **3263/5149**  **(63.4% 95%CI 62.0-64.7)** | **NS** |
| **Severe periodontitis** | **8/21**  **(38.1% 95%CI 19.0-61.3)** | **1033/5149**  **(20.1% 95%CI 19.0-21.2)** | **NS** |
| **Gingival bleeding from teeth sextants (mean sextants affected)** | **4.0** | **2.4** | **0.000** |

| **Number of bleeding sextants:** |  |  |  |
| --- | --- | --- | --- |
| **0** | **2/21**  **(9.5% 95%CI 1.7-31.8)** | **1387/5149**  **(26.9% 14.3% 95%CI 25.7-28.2)** | **0.009** |
| **1-2** | **3/21**  **(14.3% 95%CI 3.8-37.4)** | **1551/5149**  **(30.1% 14.3% 95%CI 28.9-31.4)** | **0.009** |
| **3-6** | **16/21**  **(76.2% 14.3% 95%CI 52.5-90.9)** | **2211/5149**  **(42.9% 14.3% 95%CI 41.6-44.3)** | **0.009** |

|  |  |  |  |
| --- | --- | --- | --- |

**Table S4.** The correct diagnosis of SAH and its aneurysmal etiology were confirmed by cross checking the ICD-10 codes for diagnosis with the presence of NCSP codes for intracranial aneurysm related procedures (please see Table S1 above).

| Survey participant with a diagnosis code for subarachnoid hemorrhage | ICD-10 code | NCSP code | Interpretation |
| --- | --- | --- | --- |
| 1 | I60.0 | PA2LT | Confirmed aneurysmal SAH |
| 2 | I60.9 | AAC00 | Confirmed aneurysmal SAH |
| 3 | I60.1 | - | Likely aneurysmal SAH |
| 4 | I60.1 | - | Likely aneurysmal SAH |
| 5 | I60.6 | AAC00 | Confirmed aneurysmal SAH |
| 6 | I60.3 | - | Likely aneurysmal SAH |
| 7 | I60.2 | AAC00 | Confirmed aneurysmal SAH |
| 8 | I60.6 | AAC00 | Confirmed aneurysmal SAH |
| 9 | I60.4 | AAC00 | Confirmed aneurysmal SAH |
| 10 | I60.9 | - | Possible aneurysmal SAH |
| 11 | I60.9 | - | Possible aneurysmal SAH |
| 12 | I60.9 | - | Possible aneurysmal SAH |
| 13 | I60.9 | - | Possible aneurysmal SAH |
| 14 | I60.9 | - | Possible aneurysmal SAH |
| 15 | I60.9 | - | Possible aneurysmal SAH |
| 16 | I60.9 | - | Possible aneurysmal SAH |
| 17 | I60.6 | - | Possible aneurysmal SAH |
| 18 | I60.9 | - | Possible aneurysmal SAH |
| 19 | I60.9 | - | Possible aneurysmal SAH |
| 20 | I60.1 | AAC00 | Confirmed aneurysmal SAH |
| 21 | I60.3 | PA2KT | Confirmed aneurysmal SAH |

**Table S5**. The risk factors for verified aneurysmal SAH that were identified in Cox-regression analysis including periodontal probing depth and gingival bleeding as markers of past or ongoing periodontitis and gingival infection, are given in Table 3 of the main text. Since some of the Health 2000 Survey participant may have received a diagnosis code indicating non-aneurysmal or unconfirmed aneurysmal origin (I60.9, please see Table S4 above) despite actually having had aneurysmal SAH, we also performed Cox-regression analysis with non-traumatic SAH of any kind (I60.0-I60.9) as the end event In these models, active smoking at the beginning of the follow-up and gingival bleeding on probing were the only significant predictors of SAH during follow-up. The number of followed study participants (cases) in the Cox-regression models were 2805 and 2796, with 15 of them receiving a diagnosis for non-traumatic SAH during the 13 year follow-up.

As for Table 3, smoking status was categorized as daily smoking, intermittent smoking, or not smoking. Periodontal probing depth* was categorized according to the presence of at least one tooth with ≥ 6mm probing depth (severe periodontitis), 4-5mm probing depth (periodontitis), or with no teeth having ≥4mm probing depth (no periodontitis). Gingival bleeding ** was defined as a number of tooth sextants in which bleeding occurred from the gingival margin on probing. Periodontal probing depth and gingival bleeding on probing were not included in the models simultaneously due to high intervariable correlation.

Periodontitis was 1.2 (76.2% vs 63.4%) and severe periodontitis 1.9 (38.1% vs 20.1%) times higher among those who presented with new non-traumatic SAH (I60.0-I60.9) during the follow-up, and they also had more widespread gingival bleeding compared to those without SAH, as quantitated by the number of affected sextants (2.4 (0-6) vs 4.0 (0-6), p=0.001).

# Non-smoking reduced the risk of SAH despite having periodontitis (HR 0.1, 95%CI 0.0-0.6, p=0.009

¤ Non-smoking reduced the risk of SAH despite having gingival inflammation (HR 0.1, 95%CI 0.0-0.6, p=0.005)

| **Variable** | **Cases in each group/ Number of followed subjects in the model** | | **Hazard ratio** | **95% CI** | **P-value** |
| --- | --- | --- | --- | --- | --- |
|  | |  |  |  |  |
| ***Model 1*** | |  |  |  |  |
| **Age** | |  | **1**.**021** | **0**.**969 – 1**.**074** | **0**.**439** |
| **Gender** | |  |  |  |  |
| **Male** | | **1609/2805** | **1** |  |  |
| **Female** | | **1196/2805** | **0**.**953** | **0**.**331 – 2**.**749** | **0**.**929** |
| **Hypertension** | |  |  |  |  |
| **No confirmed hypertension** | | **1677/2805** | **1** |  |  |
| **Confirmed hypertension** | | **1128/2805** | **1**.**382** | **0**.**471 – 4**.**052** | **0**.**555** |
| **Smoking status** | |  |  |  |  |
| **Non-smoking ^#^** | | **1346/2805** | **1** |  |  |
| **Intermittent smoking** | | **314/2805** | **2**.**430** | **0**.**214 – 27**.**591** | **0**.**474** |
| **Daily smoking** | | **1145/2805** | **7**.**876** | **1**.**689 – 36**.**724** | **0**.**009** |
| **Periodontitis*** | |  |  |  |  |
| **No periodontitis** | | **943/2805** | **1** |  |  |
| **Periodontitis** | | **1188/2805** | **1**.**483** | **0**.**378 – 5**.**814** | **0**.**572** |
| **Severe periodontitis** | | **674/2805** | **1**.**622** | **0**.**364 – 7**.**237** | **0**.**526** |
|  | |  |  |  |  |
| ***Model 2*** | |  |  |  |  |
| **Age** | |  | **1**.**028** | **0**.**977 – 1**.**082** | **0**.**291** |
| **Gender** | |  |  |  |  |
| **Male** | | **1605/2796** | **1** |  |  |
| **Female** | | **1191/2796** | **1**.**179** | **0**.**413 – 3**.**369** | **0**.**758** |
| **Hypertension** | |  |  |  |  |
| **No confirmed hypertension** | | **1672/2796** | **1** |  |  |
| **Confirmed hypertension** | | **1124/2796** | **1**.**450** | **0**.**494 – 4**.**255** | **0**.**499** |
| **Smoking status** | |  |  |  |  |
| **Non-smoking ¤** | | **1341/2796** | **1** |  |  |
| **Intermittent smoking** | | **314/2796** | **2**.**415** | **0**.**214 – 27**.**209** | **0**.**476** |
| **Daily smoking** | | **1141/2796** | **8**.**756** | **1**.**899 – 40**.**371** | **0**.**005** |
| **Gingival bleeding **** | |  |  |  |  |
| **0-1 sextants** | | **1211/2796** | **1** |  |  |
| **2-3 sextants** | | **725/2796** | **6**.**932** | **0**.**774 – 62**.**099** | **0**.**084** |
| **4-6 sextants** | | **860/2796** | **15**.**170** | **1**.**924 – 19**.**584** | **0**.**010** |

Fig. S1 Cox-regression showing the cumulative hazard for aneurysmal subarachnoid hemorrhage during 13 year follow-up, stratified according to smoking status at baseline. Fig A shows data for only confirmed aneurysmal SAH diagnosis and B shows the data for all diagnosis of non-traumatic SAH (I60-.0-I60.9). Separate lines are plotted according to the smoking habits. Age, gender, hypertension, and gingival inflammation are included in both regression models as co-variates in addition to the smoking status.

**A**


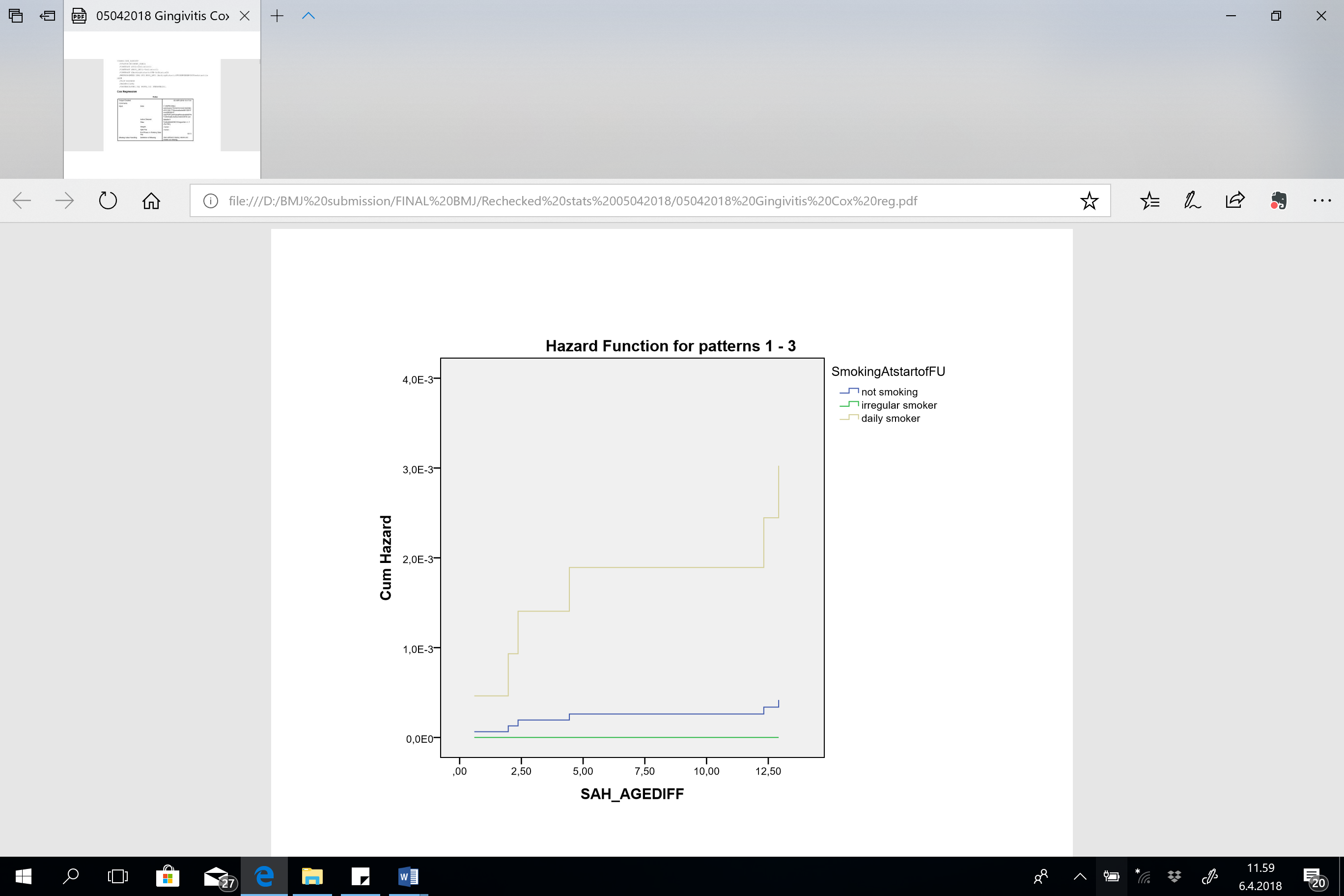


**B** **Years of follow-up**


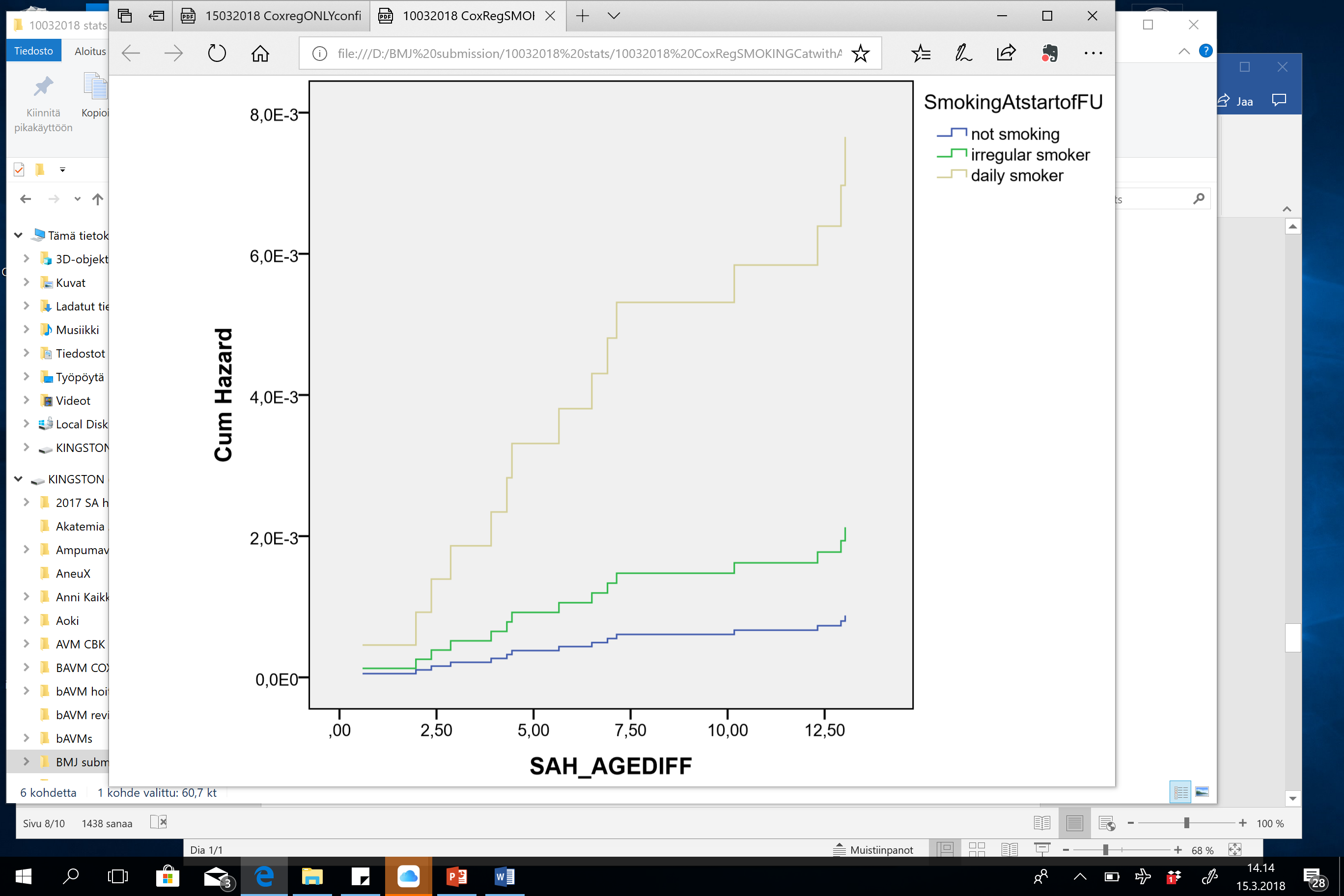


**Years of follow-up**

Figure S2. Change in periodontal status in those six patients that suffered aneurysmal SAH after baseline oral status in year 2000, and underwent a second oral examination in year 2011. Purple lines show sextants that bled on probing in the oral examination of the Health 2000 Survey (H2000), and black lines show sextants that bled in the Health 2011 (H2011) Survey. The vertical blue lines mark the borders of the teeth sextants. Depth of the gingival pockets are marked with t-bars of purple or black color for the H2000 and H2011 examinations respectively, and the missing teeth in either examination are marked with x using the same color coding.


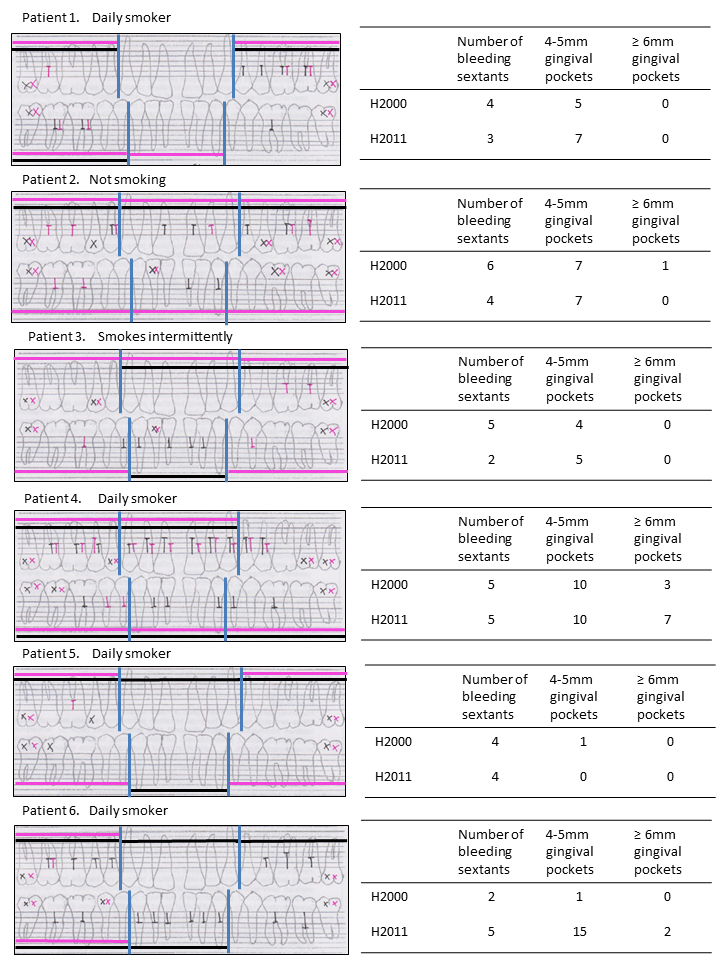


Contributions:

J.H. performed clinical oral examination of the KUH IA patients, analysed the data, and participated in the writing of the manuscript. A.L. participated in the data analysis and writing of the manuscript. J.S. supervised the clinical oral examination of KUH patients, contributed to data analysis, and participated in the writing of the manuscript. T.S. reviewed the statistical analysis performed and participated in the writing of the manuscript. A.J. contributed to data collection and reviewed the manuscript. M.N. contributed to the initial design of the study, reviewed the statistical analysis performed and participated in the writing of the manuscript. T.K. reviewed the manuscript and participated to the writing of the study. J.K. contributed to the initial design of the study, reviewed the statistical analysis performed and participated in the writing of the manuscript. P.Y. contributed to the initial design of the study, reviewed the statistical analysis performed and participated in the writing of the manuscript. AL.S. contributed to data collection, reviewed the statistical analysis performed and participated in the writing of the manuscript. J.F. designed the study, contributed to patient recruitment and data acquisition, data analysis, writing of the manuscript and overall supervision of the study
